# Nephrogenic Systemic Fibrosis in Patients with Chronic Kidney Disease after the Use of Gadolinium-Based Contrast Agents: A Review for the Cardiovascular Imager

**DOI:** 10.3390/diagnostics12081816

**Published:** 2022-07-28

**Authors:** Sebastian Gallo-Bernal, Nasly Patino-Jaramillo, Camilo A. Calixto, Sergio A. Higuera, Julian F. Forero, Juliano Lara Fernandes, Carlos Góngora, Michael S. Gee, Brian Ghoshhajra, Hector M. Medina

**Affiliations:** 1 Department of Radiology, Massachusetts General Hospital, Boston, MA 02114, USA; msgee@mgh.harvard.edu; 2 Department of Radiology, Harvard Medical School, Boston, MA 02115, USA; camilo.calixtonunez@childrens.harvard.edu; 3 Division of Cardiology, Fundacion Cardioinfantil-LaCardio, Bogota 110131, Colombia; naslygisellp@gmail.com (N.P.-J.); seanhile@hotmail.com (S.A.H.); hmedina@lacardio.org (H.M.M.); 4 Department of Radiology Boston Children’s Hospital, Boston, MA 02115, USA; 5 Division of Radiology, Fundacion Cardioinfantil-LaCardio, Bogota 110131, Colombia; jforero@lacardio.org; 6 Jose Michel Kalaf Research Institute, Radiologia Clinica de Campinas, São Paulo 13092-123, Brazil; jlaraf@terra.com.br; 7 Cardiovascular Imaging Research Center (CIRC), Division of Cardiology, Massachusetts General Hospital, Boston, MA 02114, USA; drcgongora@gmail.com (C.G.); bghoshhajra@mgh.harvard.edu (B.G.)

**Keywords:** nephrogenic systemic fibrosis, gadolinium-based contrast agents, chronic kidney disease, cardiac magnetic resonance

## Abstract

Gadolinium-enhanced cardiac magnetic resonance has revolutionized cardiac imaging in the last two decades and has emerged as an essential and powerful tool for the characterization and treatment guidance of a wide range of cardiovascular diseases. However, due to the high prevalence of chronic renal dysfunction in patients with cardiovascular conditions, the risk of nephrogenic systemic fibrosis (NSF) after gadolinium exposure has been a permanent concern. Even though the newer macrocyclic agents have proven to be much safer in patients with chronic kidney disease and end-stage renal failure, clinicians must fully understand the clinical characteristics and risk factors of this devastating pathology and maintain a high degree of suspicion to prevent and recognize it. This review aimed to summarize the existing evidence regarding the physiopathology, clinical manifestations, diagnosis, and prevention of NSF related to the use of gadolinium-based contrast agents.

## 1. Introduction to Gadolinium-Based Contrast Agents

Magnetic resonance imaging (MRI) contrast agents serve to improve diagnostic images’ sensitivity and specificity by altering the tissues’ intrinsic properties. Contrast agents carry strong paramagnetic properties that can be exploited to provide enhanced contrast between healthy and diseased tissues. By shortening the T1 and T2 relaxation times of the contiguous hydrogen nuclei, paramagnetic elements such as gadolinium (Gd) enhance the soft tissue contrast and help characterize a wide array of pathologies [1,2].

Even though elemental Gd can be toxic for humans [3], this element can be safely administered when combined with organic chelates designed to reduce the release of free Gd ions. These Gd organic chelate compounds are the basic structure of gadolinium-based contrast agents (GBCAs).

The pharmacokinetics of GBCAs helps differentiate between normal and diseased myocardium. Once administered, GBCAs diffuse rapidly out of capillaries into tissues but cannot cross intact cell membranes and equilibrate with the extracellular space. As a result, both healthy and sick myocardium accumulate GBCAs in their interstitial fluid. However, a combination of an increased volume of distribution and slower washout kinetics in sick tissues with an expanded extracellular fluid promote prolonged retention of the GBCAs, which can be detected in the late washout phase [4].

The relative accumulation of gadolinium in areas of expanded extracellular space can be seen in multiple pathologic scenarios such as fibrosis, myocardial disarray, and pathological extracellular protein infiltration [4,5]. This characteristic of the GBCAs is the key to late gadolinium enhancement (LGE), which has revolutionized cardiac magnetic resonance (CMR) in the last two decades, allowing characterization of several types of cardiomyopathies based on scar distribution.

## 2. Linear vs. Macrocyclic GBCAs

GBCAs can be classified as linear (e.g., Gd ion bridging diethylenepenta-acetic acid) or macrocyclic (e.g., a rigid, cage-like tetra-azacyclododecone compound rings Gd 3+ ions), depending on the structure that encapsulates the free Gd [5,6]. Some of the main physicochemical features of linear GBCAs (L-GBCAs) and macrocyclic GBCAs (M-GBCAs) are illustrated in Table 1 [7,8,9,10,11,12,13,14].

Intrinsic characteristics of GBCAs such as relaxivity and thermodynamic stability are described in Table 1. Relaxivity refers to the contrast’s ability to increase the surrounding water proton relaxation rate. Higher relaxivities indicate a more potent agent that requires a lower dose in order to obtain clinically useful images [12]. The hydration state of the GBCAs is the most relevant determinant of an agent’s relaxivity. By improving this parameter, it is possible to increase the clinical utility of the GBCAs [12].

On the other hand, the thermodynamic stability describes how much Gd is released at equilibrium under certain circumstances [12]. There is an inverse relationship between the complex hydration state and its thermodynamic stability [2]. As a result, a higher relaxivity decreases the thermodynamic stability of the complex, facilitating transmetalation (a process by which endogenous metals—e.g., Fe, Cu, Zn—replace Gd in the complex, freeing it from the chelate molecule) and rendering Gd more accessible to endogenous anions [12]. Transmetalation is responsible for the dissociation of GBCAs and Gd’s release in vivo.

Once Gd escapes from its organic cage, competitive binding of this metal with endogenous anions such as CO_3_^2−^ and PO_4_^3−^ promotes the formation of insoluble compounds which are free to extravasate from the bloodstream and deposit in target tissues [12,16]. In clinical practice, the release of Gd (especially by L-GBACs) increases the risk of nephrogenic systemic fibrosis (NSF) [12,17,18]. As Gd is almost exclusively cleared by the kidney, patients with chronic kidney disease (CKD) have a significantly higher risk of NSF due to the higher half-life of this metal in this population and increased risk of Gd dissociation, transmetalation, and tissue deposition.

M-GBCAs have higher thermodynamic stabilities without a significant decrease in their potency to create clinically useful images. This phenomenon can be explained by the fact they avoid the freeing of Gd ions by a lower de-chelation rate of the macrocyclic ring due to its improved molecular stability compared with linear agents [6,13].

Since their development, clinicians have shown an increased interest in M-GBCAs, such as gadobutrol and gadoterate meglumine, due to their low theoretical risk for developing NSF in patients with and without CKD and ESRD [3,19]. Their physicochemical characteristics and the experience collected during the past decades point toward a relative safety superiority of these contrast agents, given their security profile and low incidence of adverse events.

## 3. Clinical Use of GBCAs in Cardiovascular Magnetic Resonance

Gadobutrol 1.0 mmol/mL was approved for neuroimaging in January 2000 in Germany and in June 2000 in the United States. Subsequently, in the United States, it gained approval by the FDA for angiography in November 2003 [20] and CMR in 2005 [14]. GBCAs have several applications in CMR, including the characterization of a wide range of cardiomyopathies [21,22,23]. A meta-analysis of 164 studies found that the most common cardiovascular applications of GBCAs during CMR were myocardial infarction and functional testing followed by cardiomyopathies characterization. Other applications include the study of myocarditis, valvular diseases, cardiac masses, stable coronary disease, pulmonary hypertension, and right-sided heart failure [24]. Utility of CMR using gadolinium in a patient with borderline renal function is depicted in the clinical case shown next.

### Clinical Case: Use of GBCAs in a Patient with Decreased Left Ventricular Ejection Fraction and Increased Thickness

A previously healthy 73-year-old woman was admitted to the emergency room due to a 7-month history of worsening dyspnea on exertion, orthopnea, paroxysmal nocturnal dyspnea, and lower extremity without prior medical history. Physical examination showed jugular venous distention, diffuse crackles on both lung fields, and bilateral grade III pitting edema. Initial laboratories revealed anemia (Hb—10.3 g/dL; normal 13–17 gr/dL) and elevated BNP (1256 pg/mL; normal <125 pg/mL) as well as elevated lactate dehydrogenase (682 IU/L; normal 105-333 IU/L) and creatinine (1.8 mg/dL; GFR 27 mL/min/1.73 m^2^). No proteinuria was detected.

A transthoracic echocardiogram revealed thickened interatrial and interventricular septum, severe end-diastolic thickening of the left ventricle with a “granular sparkling” appearance, and a severely reduced left ventricular systolic function (LVEF) of 15%. Impaired relaxation and elevated filling pressures were consistent with severe diastolic dysfunction. A huge atrial thrombus protruding from the left appendage was noticed.

**Figure 1:**
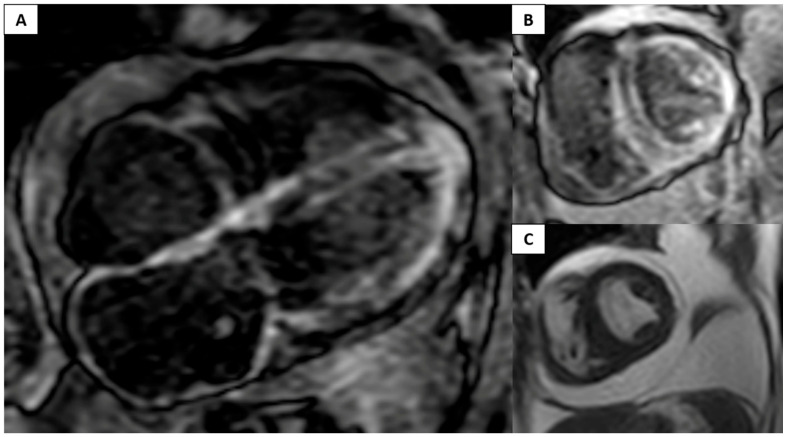
CMR in a patient with amyloidosis. (**A**) Apical-4 chamber showing diffuse LGE in the left ventricle and both atria. (**B**) Short-axis LGE with similar findings. (**C**) Short-axis cine with increased tele-diastolic thickness of the left ventricle, with pericardial and pleural effusions.

A CMR was then ordered using gadobutrol at 1 mL/kg—the latest serum creatinine was 1.5 mg/dL/GFR 34.2 mL/min/1.73 m^2^—that showed global diffuse circumferential left ventricular LGE with papillary muscle involvement (Figure 1) and extension to both atria. Cardiac amyloidosis diagnosis was considered followed by serum protein electrophoresis with no abnormal bands; however, serum immunofixation revealed a monoclonal spike determined to be IgGλ (lambda). Serum-free light chains showed an elevation in free lambda with an abnormal κ/λ ratio. A bone marrow biopsy demonstrated 1.36% of monotypic plasma cells staining for lambda light chains.

The patient was ultimately diagnosed with cardiac amyloidosis, but the family declined a percutaneous biopsy for further characterization. In the patient with CKD, the utilization of gadolinium on CMR helped determine the final diagnosis. Additionally, the were no signs of NSF at 18-month follow-up.

## 4. Overview of Nephrogenic Systemic Fibrosis

**Figure 2:**
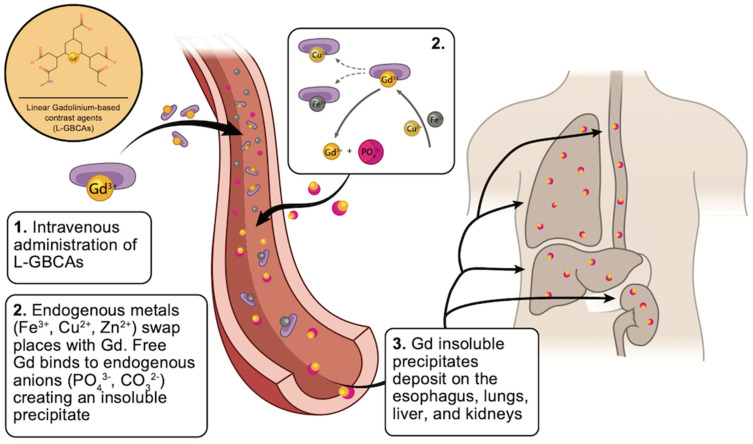
NSF pathophysiology: transmetalation and deposition of gadolinium-based contrast agents.

NSF is a multisystemic fibrotic disease that affects the skin, muscle, and other organs (including lung, esophagus, and kidney) described in patients with severe renal impairment exposed to a GBCA [3]. The pathophysiology and molecular mechanism of NSF are still a matter of debate. It is believed that the intravenous administration of some GBCAs causes a limited chelate instability that plays an essential role in the release of free Gd (Figure 2) [3,25].

**Figure 3:**
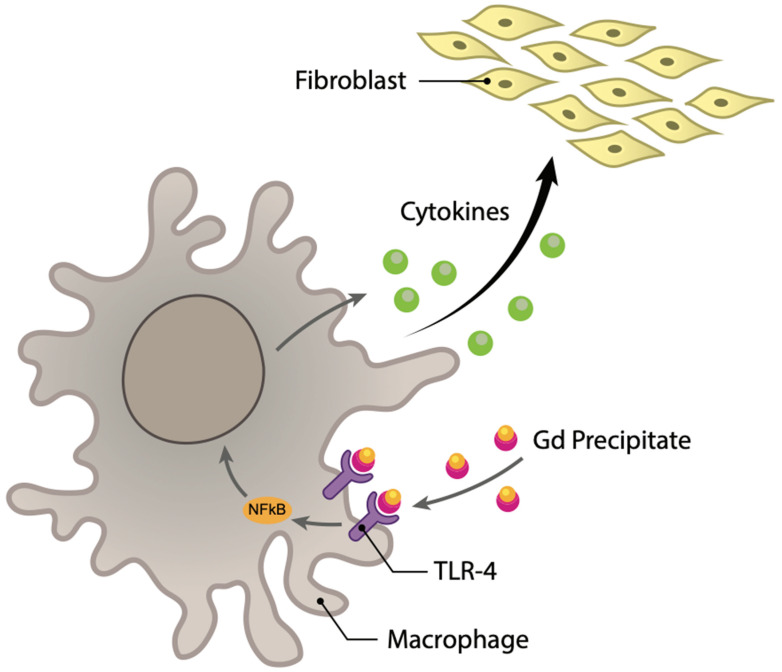
NSF: inflammatory response and subsequent systemic fibrotic reaction.

After de-chelation (by transmetalation), free Gd binds with endogenous anions, creating an insoluble precipitate that penetrates the interstitial tissue of the lung, esophagus, liver, and kidneys. In vitro studies have shown that Gd-anion complexes are highly immunogenic, binding to toll-like receptors (TLRs) on professional antigen-presenting cells (such as macrophages and dendritic cells) and leading to the release of pro-inflammatory and pro-fibrotic cytokines (Figure 3) [1].

Most GBCAs have preferential renal elimination, and their clearance is highly dependent on the glomerular filtration rate. A small percentage of the administered dose is eliminated via the hepatobiliary route. In CKD patients, the prolonged GBCAs’ half-life leads to a significant release of Gd, and therefore, a higher burden of Gd precipitates. Moreover, some of the anions that are thought to play a critical role in transmetalation (such as phosphates) are often elevated in CKD patients, facilitating this pathologic process by increasing the substrate availability for compound formation [26,27].

## 5. General Risk Factors for NSF

The main risk factor for NSF is the presence of severe acute or chronic renal insufficiency (estimated glomerular filtration rate (eGFR): 30 mL/min/1.73 m^2^) or acute renal insufficiency of any severity due to hepato-renal syndrome or in the perioperative period after liver transplantation. In 2010, the European Medicines Agency (EMA) released a statement in which NSF was considered a potential side effect of GBCAs based on the number of published reports [3]. As a result, the EMA classified the different GBCAs depending on their individual risk of triggering NSF (Table 2) based on the existing evidence and the number of reported cases [3,28]. The American College of Radiology (ACR) also proposed a similar classification system based on similar criteria (Table 3) [25]. Of note, gadobutrol and gadoterate meglumine—the most frequently used M-GBCAs—are known to confer a significantly lower risk of NSF when compared to L-GBCAs. 

Other factors that may increase the risk of NSF include multiple contrast exposures, higher cumulative doses (specially gadodiamide) [29], acidosis, hypercalcemia, hyperphosphatemia, high-dose erythropoietin therapy, hepatorenal syndrome, immunosuppression, vasculopathy, and infection [25]. Additionally, it was suggested that NSF incidence in patients on renal replacement therapy (RRT) was 19% higher in those who received the highest approved dose of any GBCAs [30].

## 6. Clinical Approach for the Diagnosis of NSF

The first description of what would be known as NSF was published in the year 2000 and consisted of a series of 15 CKD patients from different cities in the United States of America presenting with a scleroderma-like disease [31]. Five years later, additional cases of patients presenting with renal and lung compromise were described, and the term NSF was subsequently coined [32]. In 2006, the first association between NSF and GBCAs was proposed. A case series showed that from a total of nine patients with end-stage renal disease (ESRD) who underwent MR angiography with gadopentetate dimeglumine (a L-GBCA), five developed NSF [33].

Since then, multiple cases with different presentations and histopathological findings have been published, and a scoring system to identify possible NSF cases was subsequently created. Girardi et al. proposed a system based on major and minor clinical and histopathological criteria based on the Yale International NSF Registry [34]. Major criteria include patterned plaques, joint contractures, or pronounced induration (*peau d’ orange*), while minor criteria consist of linear banding, superficial plaques, dermal papules, and scleral plaques. NSF physical examination findings are summarized in Table 4 [34,35]. Histopathological criteria consist of increased dermal cellularity, CD34+ cells with tram-tracking, collagen bundles, septal involvement, and osseous metaplasia [34]. A high degree of suspicion should be maintained to detect and link these symptoms with a former GBCA exposure.

NSF usually manifests within 2–10 weeks after the initial exposure; however, clinical manifestations may only become apparent a couple of years after GBCA exposure. When inquiring about a former exposure to GBCAs, it should be categorized chronologically as acute (0–60 min), late (1 h–7 days), or very late (>7 days) in order to assess the linkage between the contrast administration and the clinical manifestations [3,38].

If NSF is suspected due to the formerly mentioned clinical and histopathological characteristics, a comprehensive review of risk factors and Gd chronological exposure should be performed. Calculation of the eGFR is vital for NSF diagnosis [3], as some degree of kidney dysfunction should be present in order to fulfill diagnostic criteria. In this way, for an accurate diagnosis, clinicians should focus their attention on the presence of a previous history of kidney transplantation, prior episodes of anuria or oliguria, a significant elevation in serum creatinine, a progressive decrease in the eGFR, or the presence of acute kidney injury (AKI) at the moment of contrast administration [3,29,34]. Such risk factors point toward the possibility of an underlying and undetected CKD, which may have prompted the onset of NSF.

Physicians should inquire for family and personal history of diseases with similar characteristics such as lipo-dermatosclerosis, chronic venous insufficiency, scleroderma, scleroderma diabeticorum, morphea, chronic graft-versus-host disease, amyloidosis, congenital fascial dystrophy, and porphyria cutanea tarda [34]. Even though these cases are rare and may have a sub-clinical course, most NSF patients have similar characteristics (especially in the initial phases) and should be excluded.

NSF severity is graded from 0–4 as follows: 0, asymptomatic; 1, mild physical, dermatologic, or neuropathic symptoms without any kind of disability; 2, moderate physical or neuropathic symptoms limiting physical performance; 3, severe symptoms limiting daily physical activities; and 4, severely disabling symptoms causing dependence on daily activities [38].

## 7. Histopathologic Examination

Histopathologic examination is essential in the definitive diagnosis of NSF. The presence of dermal hypercellularity, CD34+ cells, procollagen type I, thick and thin collagen bundles, and osseous metaplasia significantly point toward NSF diagnosis [34,35]. Additionally, some grade of fibrosis of skeletal muscle, diaphragm, heart, liver, and lung may be present and could facilitate the diagnosis (although their presence is not specific to NSF) [38].

## 8. GBCAs’ Differential Risk of NSF

As it was previously stated, when compared to L-GBCAs, M-GBCAs confer a significantly lower risk of NSF. Table 5 summarizes some of the studies assessing the safety and tolerability of M-GBCAs and L-GBCAs, especially regarding the incidence of NSF.

As an example of this differential risk between the types of GBCAs, a recent study compared a cohort of 421 patients with a 3.1% NSF incidence exposed to L-GBCAs (gadodiamide, gadopentetate dimeglumine, and gadobenate dimeglumine) versus 0% incidence in those who were exposed to a M-GBCAs (gadoteridol) [30]. Based on various retrospective reports, gadodiamide has the largest number of reported NSF cases (n = 182), followed by gadopentetate dimeglumine (n = 26), gadoversetamide (n = 5), gadoterate meglumine (n = 7), and gadobutrol (n = 3) [16,47,48]. Nevertheless, NSF cases secondary to gadobutrol are still controversial, and a clear causal association has not been established [29,43].

Gadobutrol’s safety and tolerability during contrast-enhanced MRI/angiography were evaluated in the GARDIAN study, a multicenter, international registry that included 23,708 patients [41]. The investigators concluded that gadobutrol was safe in patients with preserved kidney function and those with moderate (0.6%) or severe (0.6%) CKD. The frequency of adverse drug reactions (ADRs) was 0.7%. The most frequently reported ADRs were nausea (0.3%), followed by emesis (0.1%) and dizziness (0.1%). There were no NSF cases in the GARDIAN study after a mean follow-up of 2.8 years [41].

In a prospective, international, and multicenter study, Michaely et al. assessed the safety of gadobutrol-enhanced MRI in patients with moderate (n = 586; eGFR < 45 mL/min/1.73 m^2^) and severe (n = 284; eGFR < 30 mL/min/1.73 m^2^) CKD. A total of 927 patients was enrolled between 2008 to 2016. This study included patients with a history of organ transplantation (7.7%), hemodialysis (9.9%), diabetes (31.9%), and hypertension (58.5%) [43]. The investigators concluded that gadobutrol was safe in their patients with moderate and severe renal impairment, with no NSF cases reported after a two-year follow-up period [43].

The SECURE study assessed the safety and tolerability of gadoterate meglumine in a cohort of 35,499 patients. The total population included 514 patients that had some degree of renal impairment (eGFR less than 60 mL/min/1.73 m^2^), including 417 patients with eGFR between 30–60 mL/min/1.73 m^2^, 58 subjects with eGFR less than 30 mL/min/1.73 m^2^, and 7 with eGFR less than 15 mL/min/1.73 m^2^ on RRT. In this study, no NSF cases were observed after a 3-month follow-up period. The most frequent ADRs were urticaria (0.03%), nausea (0.02%), and emesis (0.01%) [42].

In the NSsaFe study, gadoterate meglumine was administered in 540 patients with moderate (69.4%), severe (16%), or end-stage renal impairment (12%). After a maximum follow-up of 2 years, there were no NSF reports, demonstrating gadoterate’s safety in this specific group of patients [46].

A recent meta-analysis assessed the safety of ACR group-II GBCAs in patients with stage 4 or 5 CKD (eGFR, <30 mL/min/1.73 m^2^) on RRT and concluded that the risk of NSF was less than 0.07% [19]. Interestingly, in the total population of 4931 patients included in this meta-analysis, not a single NSF case was reported secondary to group II GBCA exposure.

## 9. Prevention and Treatment of NSF

There is not a specific prophylaxis regimen to prevent the onset of NSF. The current approach is based on minimizing the impact of predisposing risk factors and performing hemodialysis sessions right after GBCA exposure in patients with a history of ESRD on RRT [35]. Hemodialysis or peritoneal dialysis should take place the same day and within 2 or 3 h after contrast administration. Hemodialysis could be more efficient than peritoneal dialysis for gadolinium clearance; however, there is insufficient evidence supporting a clinical superiority of either technique for the prevention of NSF [49] and limited evidence in the use of peritoneal dialysis to effectively remove GBCAs [50]. Maintaining adequate hydration and minimizing the concomitant exposure to nephrotoxic agents (NSAIDs, diuretics, and certain antibiotics) are also recommended, as well as not exceeding the recommended dose of administration.

Although kidney transplant improves renal function, this may not help to treat NSF [29]. Dermatologic symptoms can be treated with thalidomide, calcipotriene, and clobetasol (high-potency topical corticosteroids). Extracorporeal photopheresis improves the articulations’ range of motion and skin tightening as well. Finally, pentoxifylline demonstrated efficacy in ameliorating NSF symptoms [51].

## 10. Take-Home Messages and Clinical Applications

### 10.1. Estimation of the Glomerular Filtration Rate

- In the outpatient setting, eGFR should be estimated only in those patients with risk factors for CKD. Those patients with no risk factors or confirmed CKD should not undergo additional testing [25].
- Current evidence supports the usage of the Modification of the Diet in Renal Disease (MDRD) or the Chronic Kidney Disease Epidemiology Collaboration (CKD-EPI) in order to estimate the patient’s eGFR and base clinical decisions regarding GBCA administration [25,28,36].
- A recent creatinine value should be used (<72 h) for eGFR estimation. However, there is no evidence regarding the most appropriate timing for eGFR estimation [49].

### 10.2. Patients at Risk for Chronic Kidney Disease

- Outpatients who may be receiving GBCAs should be screened for risk factors or conditions associated with CKD [25]. This assessment should include inquiring about a history of confirmed CKD or any kidney condition (dialysis, kidney transplant, glomerulopathies, single kidney, kidney surgery, or kidney neoplasm), hypertension (requiring medical therapy), cardiovascular disease (including heart failure or coronary disease), and diabetes mellitus on metformin. For those patients identified by screening with one or more risk factors, eGFR estimation with serum creatinine should be performed [25].
- For all inpatients, eGFR should be calculated within two days before the administration of a GBCA. Additionally, the possibility of an undetected AKI should always be considered [25,49].

### 10.3. Contrast Selection

- In patients with normal kidney function (eGFR > 60 mL/min/1.73 m^2^) and no additional risk factors, the incidence of NSF after a GBCA infusion is negligible. As a result, any type of GBCAs can be safely used [43].
- In patients with stage 3 CKD (eGFR 30–50 mL/min/1.73 m^2^) and no additional risk factors, NSF’s risk is minimal. As a result, no additional actions are necessary.
- In patients with CKD stages 4 and 5 (eGFR < 30 mL/min/1.73 m^2^) or patients on RRT, ACR group-I GBCAs are contraindicated (Table 3) [25]. Only ACR group-II GBCAs should be used in this circumstance.
- Acute kidney injury: the presence of AKI significantly increases the risk of NSF [36,39,47]. In addition, the incidence of AKI is significantly higher in patients with confirmed or suspected cardiovascular disease. As a result, additional precautions should be taken into account. In AKI, there is a lag between the serum creatinine values and the actual eGFR. As a result, the sole estimation of eGFR based on creatine values could be problematic. In this setting, the ACR group-I GBCA agents should be avoided in patients with confirmed or suspected AKI [25].

### 10.4. Dialysis: Specific Recommendation

- In those patients with terminal CKD already on RRT (hemodialysis or peritoneal dialysis), dialysis should continue after receiving a GBCA. GBCA infusion should be performed as closely before hemodialysis as is possible [25]. These patients should receive dialysis the same day of the procedure, ideally 2 to 3 h after the contrast infusion to minimize the possibility of transmetalation and NSF [25,49].
- There is insufficient evidence to support changing patients from peritoneal dialysis to hemodialysis or altering dialysis prescription after the infusion of a GBCA. Peritoneal dialysis may be less effective than hemodialysis in clearing circulating GBCA; however, there is no evidence regarding the superiority of a specific type of RTT in order to decrease the risk of NSF [37,49].

### 10.5. Patients Who Require Multiple Studies

- NSF occurs most commonly in patients who received high doses of GBCA, either as a single dose or cumulatively after multiple administrations [25]. In some circumstances, patients may require multiple doses of a GBCA within a short time frame; thus, these patients are at a higher risk of developing NSF.
- In patients with preserved or moderately reduced kidney function (eGFR > 30 mL/min/1.73 m^2^), there is no contraindication if the examinations are determined to be necessary [25]. However, taking into account the elimination time of the GBCAs, it is advisable to wait at least 4 h between studies [37,49]. The usage of an ACR type-II GBCAs is advisable in this circumstance.
- In patients with residual kidney function who do not receive RRT, there should be at least 7 days between each study.
- Hemodialysis efficiently clears 70% of GBCA plasmatic concentrations after one session and more than 95% after three sessions [8,20,52]. As a result, the GBCAs’ half-life in patients on hemodialysis is similar to an individual with normal kidney function.

## 11. Limitations

- Even though current studies may not suggest NSF cases with the use of group II GBCAs, there is still epidemiological limitations to consider NSF risk as zero. There is a small number of patients with CKD stage 5 involved which underestimates the NSF incidence rate [48]. CKD patients should be assessed with a complete medical history and risk factors to determine GBCA use.
- Long-term Gd+3 brain deposition should be taken with great caution in CKD patients. This association is more frequent with the use of L-GBCAs than group II GBCA injections [48,53].
- We did not mention some other alternatives for CKD patients with eGFR < 30 mL/min/1.73 m^2^. For example, ferumoxytol is a vascular contrast agent for MR angiography with superparamagnetic properties useful to venous and arterial enhancement in stage 4 and 5 CKD patients [54].

## 12. Essentials

- Gadolinium-based contrast agents serve to improve diagnostic images’ sensitivity and specificity and characterize a wide array of cardiovascular pathologies.
- NSF is a devastating, multisystemic fibrotic disease that affects the skin, muscle, and other organs (including lung, esophagus, and kidney) described in patients with severe renal impairment exposed to a gadolinium-based contrast agent.
- There is not a specific treatment or prophylaxis regimen to treat or prevent the onset of NSF.
- Even though the newer macrocyclic agents have proven to be much safer in patients with chronic kidney disease and end-stage renal failure, clinicians must fully understand the clinical characteristics and risk factors of this devastating pathology and maintain a high degree of suspicion to prevent and recognize it. Cardiac MRI with late gadolinium enhancement (LGE) has significantly impacted the management, decision making, and diagnosis of various cardiomyopathy or interstitial heart disease. However, the over-concerned about nephrogenic systemic fibrosis may make cardiac MRI with LGE be avoided inappropriately. The risk and benefits of this imaging study should be balanced.

## Figures and Tables

**Table 1:** Physicochemical characteristics of gadolinium-based contrast agents [7,8,9,10,11,12,13,14].

Molecule, Trade Name, and (Vendor)	Type	Osmolality (mOsm/kg H_2_O) *	Viscosity (mPa•) *	Thermodynamic Complex Stability (log Keq)	Relaxivities r1/r2 in Plasma (mmol/L) at 1.5 T *	Concentration (mol/L)	Approved Intravenous Dose (mmol/kg)	Comments
*Gadobutrol*, Gadavist (Bayer Healthcare)	Macrocyclic Non-ionic	1603	4.96	21.8	5.2/6.1	1	0.1–0.3	Highest viscosity. It is marketed as Gadovist outside the United States.
*Gadoterate meglumine*, Dotarem (Guerbet)	Macrocyclic Ionic	1350	2.4	25.6	3.6/4.3	0.5	0.1–0.2	In 2019, a generic version of Dotarem was introduced (Clariscan, GE Healthcare). Some animal studies have shown slightly higher levels of gadolinium deposition with Clariscan compared to Dotarem [15].
*Gadoteridol*, ProHance (Bracco)	Macrocyclic Non-ionic	630	1.3	23.8	4.1/5	0.5	0.1–0.2	Lowest viscosity and osmolality. Below average viscosity.
*Gadopentetate dimeglumine*, Magnevist (Bayer Healthcare)	Linear Ionic	1960	1.9	22.1	4.1/4.6	0.5	0.1–0.3	Oldest approved agent. Below average relaxivity. High risk of NSF.
*Gadodiamide*, Omniscan (GE Healthcare)	Linear Non-ionic	783	1.4	16.9	4.3/5.2	0.5	0.1–0.3	Low thermodynamic stability; very high risk of NSF. Use suspended in the European Union.
*Gadobenate dimeglumine*, MultiHance (Bracco)	Linear Ionic	1970	5.3	22.6	6.3/8.7	0.5	0.05–0.1	Highest relaxivity of extracellular GBCAs. EMA restricted to hepatobiliary imaging.
*Gadoxetate disodium*, Eovist/Primovist (Bayer Healthcare)	Linear Ionic	688	1.19	23.5	6.9/8.7	0.25	0.025	Designed for liver imaging. Renal and biliary excretion. Very high relaxivity. EMA restricted to hepatobiliary imaging.

* At 37 °C; NSF: nephrogenic systemic fibrosis; GBCA: gadolinium-based contrast agent; EMA: European Medicines Agency.

**Table 2:** EMA classification for NSF risk among GBCAs ^§^.

High Risk	Intermediate Risk	Low Risk
Gadodiamide	Gadobenate dimeglumine	Gadobutrol
Gadoversetamide	Gadoxetate disodium	Gadoteridol
Gadopentetate dimeglumine	Gadofosvest	Gadoterate meglumine

§^:^ Adapted with permission from: European Medicines Agency: Assessment report for Gadolinium-containing contrast agents; Reference: [10]. 2022, European Medicines Agency.

**Table 3:** ACR Manual Classification of Gadolinium-Based Agents Relative to NSF ^†^.

ACR Group I *	ACR Group II **	ACR Group III ***
Gadodiamide Gadoversetamide Gadopentetate dimeglumine	Gadobenate dimeglumine	Gadoxetate disodium
	Gadobutrol	
	Gadoteric acid	
	Gadoteridol	

^†:^ Adapted with permission from: ACR Committee on Drugs and Contrast Media. ACR Manual on Contrast Media; Reference: [25]. 2022, American College of Radiology. *: Agents associate with the greatest number of NSF cases. **: Agents associated with few, if any, unconfounded NSF cases. ***: Agents for which data remain limited regarding NSF risk, but for which few, if any, unconfounded cases of NSF have been reported.

**Table 4:** Signs and symptoms of NSF [7,27,29,34,36,37].

Clinical History	Signs	Symptoms
L-GBCA exposure (2–8 weeks—10 years after gadolinium uptake) **Family history of NSF Renal:** AKI, history of chronic kidney disease, kidney transplantation, or hemodialysis	**Eye:** Whitish-yellow plaques with vascular ectasia **Skin changes:** Hyperpigmentation, symmetrical lesions, rash-patterned plaques (red to violaceous lesions), superficial papules (beefy lesions in upper extremities), macules, nodules, skin thickening (cobble stoning or peau d’orange appearance) **Renal:** Volume overload, uremia **Extremities:** Limited range motion, joint contractures (finger, elbows, toes, and ankles), symmetric edema (inferior limbs)	**Eye:** Vision impairment, conjunctival injection, and white-yellow scleral plaques **Skin:** Pruritus, burning pain, new skin lesion, induration, and swelling **Extremities:** Edema, pain, and decreased mobility of the joints **Urinary findings:** Anuria, oliguria

**Table 5:** Safety and tolerability studies of M-GBCAs and L-GBCAs.

Authors, Study Name	Year	Study Type	Total Number of Patients	GBCA	Number of Patients with Renal Impairment	NSF Cases at Maximum Follow-Up	Estimated NSF Incidence
Aneet et al. [39]	2007	Retrospective cohort	467 (87 with gadolinium exposure)	Gadopentetate diglumine (L) and gadodiamide (L)	87 patients with end-stage renal disease (patients in dialysis)	3	4.3 cases per 1000 patients-year (overall NSF incidence)
Wang Y et al. [40]	2011	Retrospective cohort	52,954 (after the 2007 Restrictive GBCA guidelines were implemented)	Gadopentetate diglumine (L) and gadobenate diglumine (L)	6454 patients with GFR between 30–59 mL/min/m^2^; 36 patients with GFR lower than 30 mL/min/m^2^	0	-
Prince MR et al. GARDIAN study [41]	2016	Prospective cohort	23,708	Gadobutrol (M)	100 patients with moderate renal impairment (GFR: 30–59 mL/min/1.73 m^2^) and 31 patients with severe renal impairment (<30 mL/min/1.73 m^2^)	0	-
Soyer P et al. SECURE study [42]	2017	Prospective cohort	35,499	Gadorate meglumine (M)	417 patients with moderate renal impairment (GFR: 30–59 mL/min/1.73 m^2^); 58 patients with severe renal impairment (GFR: 15–39 mL/min/1.73 m^2^); 7 patients with end-stage renal impairment (GFR: <15 mL/min/1.73 m^2^) or dialysis.	0	-
Michaely HJ et al. GRIP study [43]	2017	Prospective cohort	908	Gadobutrol (M)	586 with moderate (GFR: 30–59 mL/min/1.73 m^2^) and 284 with severe renal impairment (<30 mL/min/1.73 m^2^)	0	-
Tsushima Y et al. [44]	2018	Prospective cohort	3337	Gadobutrol (M)	356 patients with GFR between 45–59 mL/min/m^2^; 71 patients with GFR between 30–44 mL/min/m^2^; 4 patients with GFR between 15–29 mL/min/m^2^; 1 patient with GFR < 15 mL/min/m^2^	0	-
Young LK [45]	2019	Retrospective cohort	22,897	Gadorate meglumine (M)	2570 patients with moderate renal impairment (GFR: 30–59 mL/min/1.73 m^2^); 464 patients with severe renal impairment (GFR: 15–39 mL/min/1.73 m^2^); 123 patients with end-stage renal impairment (GFR: <15 mL/min/1.73 m^2^) or dialysis.	0	-
McWilliams RG et al. NSsaFe study [46]	2020	Prospective cohort	540	Gadorate meglumine (M)	226 patients with moderate renal impairment (GFR: 30–59 mL/min/1.73 m^2^); 59 patients with severe renal impairment (GFR: 15–39 mL/min/1.73 m^2^); 58 patients with end-stage renal impairment (GFR: <15 mL/min/1.73 m^2^) or dialysis.	0	-

M: macrocyclic gadolinium-based contrast agent. L: linear gadolinium-based contrast agent.
